# Supplementary material for: Characteristics and Injury Patterns of Road Traffic Injuries in Urban and Rural Uganda—A Retrospective Medical Record Review Study in Two Hospitals
Source: Int J Environ Res Public Health. 2021 Jul 19;18(14):7663. doi: 10.3390/ijerph18147663 (PMC8304504; doi:10.3390/ijerph18147663)
Supplement: Supplementary file 1 [file ijerph-18-07663-s001.zip › ijerph-1266742-supplementary.pdf]

Supplementary

Figure S1: Interface for data collection and during the on-site Medical Record Review

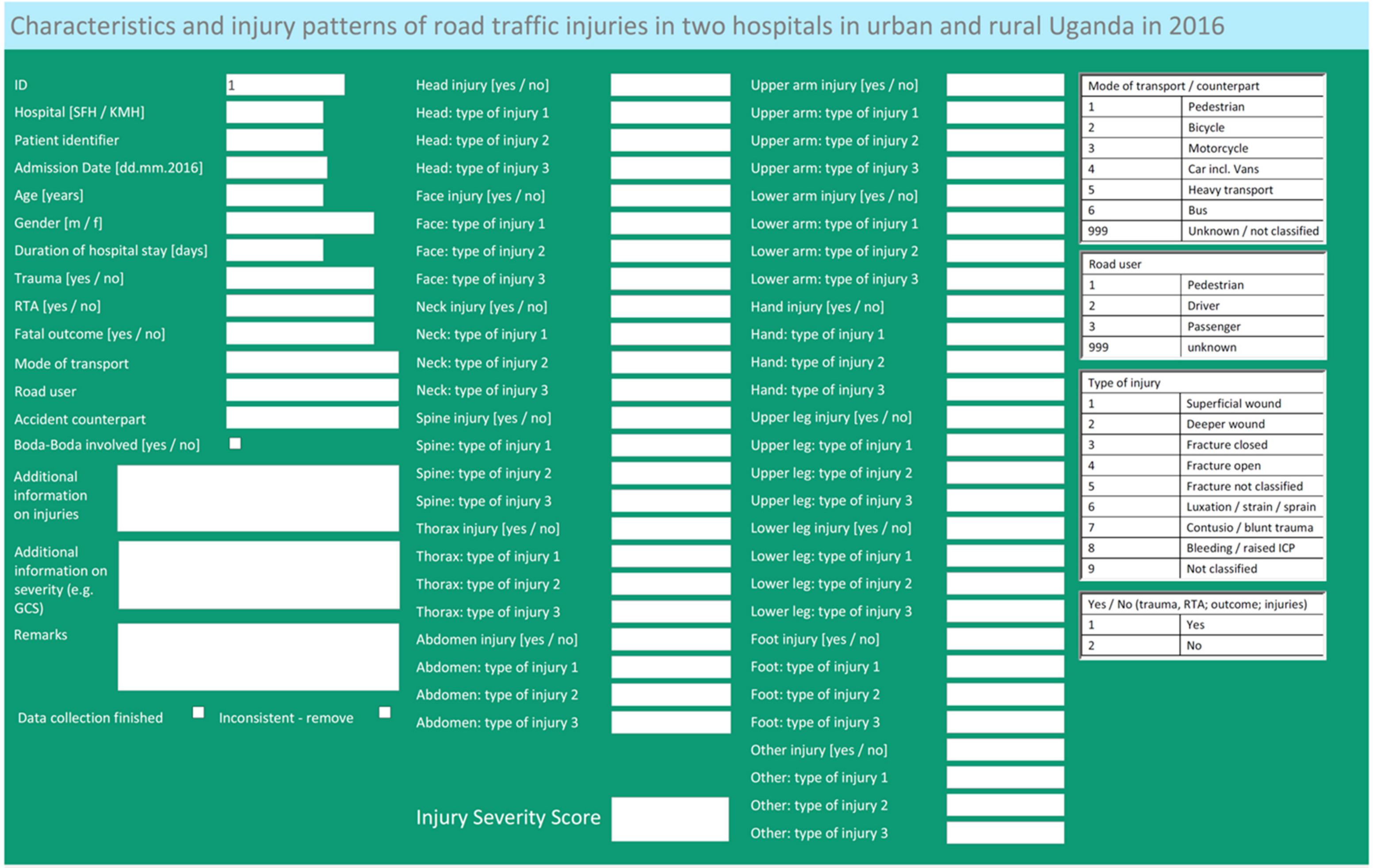

Characteristics and injury patterns of road traffic injuries in two hospitals in urban and rural Uganda in 2016

ID: 1
Hospital [SFH / KMH]
Patient identifier
Admission Date [dd.mm.2016]
Age [years]
Gender [m / f]
Duration of hospital stay [days]
Trauma [yes / no]
RTA [yes / no]
Fatal outcome [yes / no]
Mode of transport
Road user
Accident counterpart
Boda-Boda involved [yes / no]
Additional information on injuries
Additional information on severity (e.g. GCS)
Remarks
Data collection finished
Inconsistent - remove

Head injury [yes / no]
Head: type of injury 1
Head: type of injury 2
Head: type of injury 3
Face injury [yes / no]
Face: type of injury 1
Face: type of injury 2
Face: type of injury 3
Neck injury [yes / no]
Neck: type of injury 1
Neck: type of injury 2
Neck: type of injury 3
Spine injury [yes / no]
Spine: type of injury 1
Spine: type of injury 2
Spine: type of injury 3
Thorax injury [yes / no]
Thorax: type of injury 1
Thorax: type of injury 2
Thorax: type of injury 3
Abdomen injury [yes / no]
Abdomen: type of injury 1
Abdomen: type of injury 2
Abdomen: type of injury 3
Injury Severity Score

Upper arm injury [yes / no]
Upper arm: type of injury 1
Upper arm: type of injury 2
Upper arm: type of injury 3
Lower arm injury [yes / no]
Lower arm: type of injury 1
Lower arm: type of injury 2
Lower arm: type of injury 3
Hand injury [yes / no]
Hand: type of injury 1
Hand: type of injury 2
Hand: type of injury 3
Upper leg injury [yes / no]
Upper leg: type of injury 1
Upper leg: type of injury 2
Upper leg: type of injury 3
Lower leg injury [yes / no]
Lower leg: type of injury 1
Lower leg: type of injury 2
Lower leg: type of injury 3
Foot injury [yes / no]
Foot: type of injury 1
Foot: type of injury 2
Foot: type of injury 3
Other injury [yes / no]
Other: type of injury 1
Other: type of injury 2
Other: type of injury 3

Mode of transport / counterpart	
1	Pedestrian
2	Bicycle
3	Motorcycle
4	Car incl. Vans
5	Heavy transport
6	Bus
999	Unknown / not classified

Road user	
1	Pedestrian
2	Driver
3	Passenger
999	unknown

Type of injury	
1	Superficial wound
2	Deeper wound
3	Fracture closed
4	Fracture open
5	Fracture not classified
6	Luxation / strain / sprain
7	Contusio / blunt trauma
8	Bleeding / raised ICP
9	Not classified

Yes / No (trauma, RTA; outcome; injuries)	
1	Yes
2	No

Table S1: Proportion of incomplete Medical Reviews for mode of transportation, road user and crash counterpart in the urban and rural hospital

Data incomplete for:	Urban Hospital 1	Rural Hospital 2
**Mode of transportation**	7.2%	47.6%
**Road user**	9.2%	52.0%
**Crash counterpart**	35.5%	72.1%

Table S1: Calculation of the Injury Severity Score (ISS) – adapted to MRR with limited information

Injury location	Injury type	severity
**Head**	Contusion without loss of consciousness (LOC) / "mild closed head injury (CHI)"	1
**Head**	Contusion with LOC < 15 min / un-displaced skull or facial bone fracture / "moderate CHI"	2
**Head**	Closed head injury (with radiological evidence) / displaced closed skull fracture without LOC / "severe CHI"	3
**Head**	Open head injury with (radiological) signs of intracranial injury	4
**Head**	Bleeding / oedema / pneumocranium / raised intracranial pressure (ICP)	5
**Face**	Dislocation of teeth	1
**Face**	Fractures of facial bones (single / multiple)	2 / 3
**Neck**	Contusion / blunt trauma	1
**Neck**	Cervical fracture without cord damage	3
**Neck**	Cervical spine injury with partial paralysis	4
**Neck**	Cervical spine injury with quadriplegia	5
**Thorax**	Contusion / blunt trauma	1
**Thorax**	Rib fracture (single / multiple)	2 / 3
**Thorax**	Bleeding / haematothorax / rupture of diaphragm	3
**Thorax**	Cardiac arrest	5
**Abdomen**	Contusion / blunt trauma	1
**Abdomen**	Kidney haematoma	2
**Abdomen**	Urethral laceration / spleen rupture / bladder injury	3
**Abdomen**	Bleeding / haematoperitoneum	4
**Extremity and pelvis**		
	**Fractures**<br>**Clavicle / radius / scapula / single digits**<br>**Clavicle + scapula / radius + ulna / tibia + fibula / humerus**<br>**Femur / Pelvis / multiple fractures**<br>**Multiple long-bone (e.g. both legs)**	 1<br>2<br>3<br>4
	**Dislocations**<br>**Elbow / digits**<br>**Shoulder / knee**<br>**Pelvis / femur**	 1<br>2<br>3
	**Laceration of major nerves / vessels of extremities**	3
	**Amputation of limbs**	4
	**Multiple open limb fractures**	5

**External**		
**All**	Superficial wound / abrasion	1
**All**	Open / deeper wound / laceration / open fracture	2
**All**	Multiple deep open wounds (≥ 3) / “de-gloving” wounds	3
**All**	Severe lacerations with dangerous haemorrhage	4
**All**	Burns 1°	1
**All**	Burns 2°/3° involving 10-20% BSA	2

Table S2: Descriptive analysis

Variables	Levels and values calculated	Descriptive statistics						
**Demographic data**								
		**Urban Hospital 1**		**Rural Hospital 2**		**combined**		**Significance** (p; *test utilized*)
**AGE** (Continuous / ordered categorical)								*Mann-Whitney*
	Mean [Years]	29.21		31.68		30.67		**0.018**
	Mean for no-RTC	26.93		31.29		29.59		**0.004**
	Mean for RTC	30.83		32.0		31.50		0.499
	Age groups [n / %]							
	0 – 4	54	7.8	44	4.4	98	5.8	*Chi-squared:*
	5 – 14	70	10.2	97	9.7	167	9.9	**0.003**
	15 – 24	150	21.8	215	21.6	365	21.7	0.774
	25 – 34	208	30.2	304	30.6	512	30.4	0.924
	35 – 44	94	13.7	136	13.7	230	13.7	0.888
	45 – 54	56	8.1	88	8.8	144	8.6	0.997
	55 – 64	25	3.6	39	3.9	64	3.8	0.611
	> 65	31	4.1	72	7.2	103	6.1	0.763
								**0.022**
**GENDER** (Categorical, nominal)	**all trauma**							*Chi-squared:*
	n / % Male	500	72.7	746	75.0	1,246	74.0	0.290
	n / % Female	188	27.3	249	25.0	437	26.0	
	**RTC**							*Chi-squared:*
	n / % Male	278	69.0	407	74.3	685	72.0	0.073
	n / % Female	125	31.0	141	25.7	266	28.0	
	**no-RTC**							*Chi-squared:*
	n / % Male	222	77.9	339	75.8	561	76.6	0.522
	n / % Female	63	22.1	108	24.2	171	23.4	
**Hospital admission data**								
		**Urban Hospital 1**		**Rural Hospital 2**		**combined**		**Significance** (p; *test utilized*)
**HOSPITAL**	Total	5,018		9,735		14,753		

**ADMISSIONS DURING STUDY PERIOD**<br><br>**(w/o maternity ward)**<br>**(Categorical, ordinal)**	Prevalence trauma<br>Prevalence of RTC from total Admissions<br><br>**Prevalence of RTC from all trauma admissions**	688<br>403<br><br>58.6%	13.7<br>8.0	995<br>548<br><br>55.1%	10.2<br>5.6	1,683<br>951<br><br>56.5%	11.4<br>6.4	*Chi-squared:*<br>0.154
**TRAUMA MORTALITY**<br><br>**(Categorical, ordinal)**	**no-RTC**	20 no-RTC trauma	7.0	13 no-RTC cases	2.9	33 no-RTC cases	4.5	*Chi-squared:*<br>**0.009**
	**RTC**	17 RTC trauma	4.0	17 RTC cases	3.1	34 RTC cases	3.6	0.360
	**Total number of fatal cases**	37 of all trauma cases	5.4	30 of all trauma cases	3.0	67 of all trauma cases	4.0	**0.015**
**DURATION OF HOSPITAL STAY**<br><br>**(Continuous / ordered categorical)**	**all trauma**<br>[days]<br><br>Short (0-3 days)<br>Moderate (4-9 days)<br>Long (> 10 days)	Mean: 5.0<br>Median: 3.0<br><br>353<br>262<br>73	 <br><br>51.3<br>38.1<br>10.6	Mean: 7.1<br>Median: 4.0<br><br>435<br>379<br>181	 <br><br>43.7<br>38.1<br>18.2	Mean: 6.3<br>Median: 4.0<br><br>788<br>641<br>254	 <br><br>46.8<br>38.1<br>15.1	*Mann-Whitney U*<br>**< 0.001**<br><br>*Kruskal-Wallis*<br>**< 0.001**
	**RTC**<br>[days]<br><br>Short (0-3 days)<br>Moderate (4-9 days)<br>Long (> 10 days)	Mean: 4.92<br>Median: 3.0<br><br>207<br>153<br>43	 <br><br>51.4<br>38.0<br>10.7	Mean: 7.9<br>Median: 5.0<br><br>215<br>218<br>115	 <br><br>39.2<br>39.8<br>21.0	Mean: 6.7<br>Median: 4.0<br><br>422<br>371<br>158	 <br><br>44.4<br>39.0<br>16.6	*Mann-Whitney U*<br>**< 0.001**<br><br>*Kruskal-Wallis*<br>**< 0.001**
	**no-RTC**<br>[days]<br><br>Short (0-3 days)<br>Moderate (4-9 days)<br>Long (> 10 days)	Mean: 5.13<br>Median: 3.0<br><br>146<br>109<br>30	 <br><br>51.2<br>38.2<br>10.5	Mean: 6.11<br>Median: 4.0<br><br>220<br>161<br>66	 <br><br>49.2<br>36.0<br>14.8	Mean: 5.73<br>Median: 3.50<br><br>366 / 50.0%<br>270 / 36.9%<br>96 / 13.1%		*Mann-Whitney U*<br>0.681<br><br>*Kruskal-Wallis*<br>0.330
**RTC characteristics**								

		Urban Hospital 1	Rural Hospital 2	combined	Significance (p; *test utilized*)
					Chi-squared:
**MODE OF TRANSPORT** (Categorical, ordinal)	Pedestrian	139 / 37.2%	84 / 29.3%	223 / 33.7%	**p = 0.033**
	Bicycle	8 / 2.1%	10 / 3.5%	18 / 2.7%	p = 0.292
	Motorcycle	171 / 45.7%	157 / 54.7%	328 / 49.6%	**p = 0.022**
	Car (including vans)	38 / 10.2%	26 / 9.1%	64 / 9.7%	p = 0.635
	Heavy transport vehicle	13 / 3.5%	6 / 2.1%	19 / 2.9%	p = 0.291
	Bus	5 / 1.3%	4 / 1.4%	9 / 1.4%	p = 0.950
	Others / unknown*	29 / 403 (7.2%)	261 / 548 (47.6%)	290 / 951 (30.5%)	
**ROAD USER** (Categorical, ordinal)					Chi-squared Test: (others excluded)
	Pedestrian	139 / 38%	84 / 35.5%	223 / 35.5%	p = 0.118
	Driver	102 / 27.9%	58 / 22.1%	160 / 25.4%	p = 0.099
	Passenger	125 / 34.2%	121 / 46.0%	246 / 39.1%	**p = 0.003**
	Unknown	37 / 403 (9.2%)	285 / 548 (52%)	322 / 951 (33.9%)	
					Chi-squared Test: (others excluded)
**Crash COUNTERPART** (Categorical, ordinal)	Pedestrian	7 / 2.7%	1 / 0.7%	8 / 1.9%	p = 0.147
	Bicycle	5 / 1.9%	3 / 2.0%	8 / 1.9%	p = 0.979
	Motorcycle	81 / 31.2%	92 / 60.1%	173 / 41.9%	**p < 0.001**
	Car (including vans)	147 / 56.5%%	52 / 34.0%	199 / 48.2%	**p < 0.001**
	Heavy transport vehicle	16 / 6.2%	5 / 3.3%	21 / 5.1%	p = 0.197
	Bus	4 / 1.5%	0 / 0%	4 / 1.0%	p = 0.123
	Others / unknown*	143 / 403 (35.5%)	395 / 548 (72.1%)	538 / 951 (56.6%)	
**Injury characteristics**					
		**Urban Hospital 1**	**Rural Hospital 2**	**combined**	**Significance** (p; *test utilized*)
**INJURY SEVERITY SCORE (ISS)**		Mean: 13.79 Median: 10.0	Mean: 7.1 Median: 5.0	Mean: 9.9 Median: 8.0	*Mann-Whitney U* **p < 0.001**

(Continuous / ordered categorical)	Mild (ISS < 9)<br>Moderate (ISS 9-15)<br>Severe (ISS 16-25)<br>Profound (ISS > 25)	163 / 40.4%<br>79 / 19.6%<br>98 / 24.3%<br>63 / 15.6%	358 / 65.4%<br>142 / 26.0%<br>41 / 7.5%<br>6 / 1.1%	521 / 54.8%<br>221 / 23.3%<br>129 / 14.6%<br>69 / 7.3%	Kruskal-Wallis Test<br>**p < 0.001**
**INJURY LOCATION**<br><br>**(Categorical, ordinal)**					*Chi-squared:*
	Head	259 / 64.3%	140 / 25.5%	399 / 42.0%	**p < 0.001**
	Face	111 / 27.5%	142 / 25.9%	253 / 26.6%	p = 0.558
	Neck	0 / 0%	2 / 0.4%	2 / 0.2%	p = 0.225
	Spine	13 / 3.2%	6 / 1.1%	19 / 2.0%	**p = 0.020**
	Head/face/neck/spine (combined)	284 / 70.5%	239 / 43.6%	523 / 55.0%	**p < 0.001**
	Thorax	56 / 13.9%	45 / 8.2%	101 / 10.6%	**p = 0.005**
	Abdomen	31 / 7.7%	13 / 2.4%	44 / 4.6%	**p < 0.001**
	Thorax / Abdomen (combined)	77 / 19.1%	58 / 10.6%	135 / 14.2%	**p < 0.001**
	Shoulder / upper arm	68 / 16.9%	76 / 13.9%	144 / 15.1%	p = 0.201
	Elbow / lower arm	27 / 6.7%	56 / 10.2%	83 / 8.7%	p = 0.057
	Hand wrist / hand	21 / 5.2%	17 / 3.1%	38 / 4.0%	p = 0.101
	upper extremity (combined)	104 / 25.8%	139 / 25.4%	243 / 25.6%	p = 0.877
	Hip / femur	65 / 16.1%	128 / 23.4%	193 / 20.3%	**p = 0.006**
	Knee / lower leg	74 / 18.4%	175 / 31.9%	249 / 26.2%	**p < 0.001**
	Ankle / foot	26 / 6.5%	39 / 7.1%	65 / 6.8%	p = 0.688
	lower extremity (combined)	129 / 32.0%	293 / 53.5%	422 / 44.4%	**p < 0.001**
	all extremities (combined)	197 / 48.9%	389 / 71.0%	586 / 61.6%	**p < 0.001**
	Others / not classified	57 / 14.1%	48 / 8.8%	105 / 11.0 %	(p = 0.01)
**MULTIPLE INJURY LOCATIONS**					*Chi-squared*
	1	147 / 36.5%	298 / 54.4%	445 / 46.8%	**p < 0.001**
	2	148 / 36.7%	174 / 31.8%	322 / 33.9%	p = 0.109
	3	78 / 19.4%	65 / 11.9%	143 / 15.0%	**p = 0.001**
	4	22 / 5.5%	9 / 1.6%	31 / 3.3%	**p = 0.001**
	5	5 / 1.2%	2 / 0.4%	7 / 0.7%	p = 0.118
	6	3 / 0.7%	0 / 0%	3 / 0.3%	**p = 0.043**
**INJURY TYPE**					*Chi-squared*

(Categorical, ordinal)	Superficial wound (e.g. abrasion)	142 / 35.2%	181 / 33.0%	323 / 34.0%	p = 0.478
	Open (deeper) wound (e.g. laceration)	124 / 30.8%%	229 / 41.8%	353 / 37.1%	**p = 0.001**
	STI (TOTAL)	232 / 57.6%	373 / 68.1%	605 / 63.6%	**p = 0.001**
	Fracture (closed)	17 / 4.2%	52 / 9.5%	69 / 7.3%	**p = 0.002**
	Fracture (open)	23 / 5.7%	71 / 13.0%	94 / 9.9%	**p < 0.001**
	Fracture (not classified)	222 / 55.1%	201 / 36.7%	423 / 44.5%	**p < 0.001**
	Fractures (TOTAL)	249 / 61.8%	308 / 56.2%	557 / 58.6%	p = 0.084
	Fractures (TOTAL without head)	186 / 46.2%	300 / 54.7%	486 / 51.1%	**p = 0.009**
	Fractures (only head)	88 / 21.8%	10 / 1.8%	98 / 10.3%	**p < 0.001**
	Fractures upper leg	45 / 11.2%	107 / 19.5%	152 / 16.0%	**p = 0.001**
	Fractures lower leg	62 / 15.4%	126 / 23.0%	188 / 19.8%	**p = 0.004**
	Luxation / sprain / strain	18 / 4.5%	29 / 5.3%	47 / 4.9%	p = 0.562
	Contusion / blunt trauma	296 / 73.4%	139 / 25.4%	435 / 45.7%	**p < 0.001**
	bleeding / raised intracranial pressure	135 / 33.5%	10 / 1.8%	145 / 15.2%	**p < 0.001**
	Others / not classified	0 / 0%	1 / 0.2%	1 / 0.1%	p = 0.391
**MULTIPLE INJURIES**	Number of all injuries in one patient				Chi-squared
	1	55 / 13.6%	217 / 39.6%	272 / 28.6%	**p < 0.001**
	2	121 / 30.0%	192 / 35.0%	313 / 32.9%	p = 0.104
	3	114 / 28.3%	102 / 18.6%	216 / 22.7%	**p < 0.001**
	4	62 / 15.4%	28 / 5.1%	90 / 9.5%	**p < 0.001**
	5	30 / 7.4%	6 / 1.1%	36 / 3.8%	**p < 0.001**
	6	15 / 3.7%	3 / 0.5	18 / 1.9%	**p < 0.001**
	7	3 / 0.7%	0 / 0%	3 / 0.3%	**p = 0.043**
	8	2 / 0.5%	0 / 0%	2 / 0.2%	p = 0.099
	9	1 / 0.2%	0 / 0%	1 / 0.1%	p = 0.243
	Number of fractures in one patient (total)				Chi-squared
	0	154 / 38.2%	240 / 43.8%	394 / 41.4%	p = 0.084
	1	179 /44.4%	267 /48.7%	446 / 46.9%	p = 0.189
	2	59 / 14.6%	38 / 6.9%	97 / 10.2%	**p < 0.001**
	3	11 / 2.7%	3 / 0.5%	14 / 1.5%	**p = 0.006**

	Number of fractures in one patient (without head)<br>0<br>1<br>2<br>3	 <br><br>217 / 53.8%<br>136 / 33.7%<br>44 / 10.9%<br>6 / 1.5%	 <br><br>248 / 45.3%<br>260 / 47.4%<br>38 / 6.9%<br>2 / 0.4%	 <br><br>465 / 48.9%<br>396 / 41.6%<br>82 / 8.6%<br>8 / 0.8%	 <br>Chi-squared:<br>**p = 0.009**<br>**p < 0.001**<br>**p = 0.031**<br>p = 0.061
	Number of STI in one patient<br>0<br>1<br>2<br>3<br>4<br>5	 <br>171 / 42.4%<br>153 / 38.0%<br>53 / 13.2%<br>22 / 5.5%<br>3 / 0.7%<br>1 / 0.2%	 <br>175 / 31.9%<br>253 / 46.2%<br>92 / 16.8%<br>25 / 4.6%<br>3 / 0.5%<br>0 / 0%	 <br>346 / 36.4%<br>406 / 42.7%<br>145 / 15.2%<br>47 / 4.9%<br>6 / 0.6%<br>1 / 0.1%	 Chi-squared<br>**p = 0.001**<br>**p = 0.012**<br>p = 0.123<br>p = 0.528<br>p = 0.705<br>p = 0.243
